# Supplementary material for: Sign language use in healthcare: professionals’ insight
Source: PeerJ. 2025 Jun 2;13:e19446. doi: 10.7717/peerj.19446 (PMC12139612; doi:10.7717/peerj.19446)
Supplement: Supplemental Information 2 — There was no separate consent form provided to the participants, however, there was a statement at the top of the survey informs the participants about the nature of the study. They were informed that they could choose not to participate and that proceeding on the survey confirms their agreement to participate. [file peerj-13-19446-s002.docx]

**اسمي أميمة قاضي باحثة في كلية التمريض بجامعة الملك سعود في الرياض، أعمل على بحث يهدف إلى قياس**

**توجه الممارسين الصحيين العاملين بالقطاع الصحي في المستشفيات الحكومية والخاصة ومراكز الرعاية الأولية في المملكة العربية السعودية اتجاه تعلم واستخدام لغة الإشارة كوسيله للتواصل مع المرضى أو ذويهم من فئة الصم وضعاف السمع.**

**إن كنت ممارس/ه صحي يعمل في أحد قطاعات الصحة المذكورة بالأعلى فإنه يحق لك المشاركة في هذا البحث.**

**تتم المشاركة في هذا البحث عن طريق الإجابة على الاستبانة أدناه والذي قد يستغرق ما بين (15 إلى 20) خمسة عشر إلى عشرون دقيقه.**

المشاركة في البحث تطوعيه وإرادية ولا تترتب عليها أي حوافز ماديه أو عينيه. يمكنك الانسحاب في أي لحظه تشعر/ين بذلك دون أن يترتب على ذلك تحمل أي مسؤوليه.

لن يتم طلب أي معلومات توحي إلى شخصية المشارك والتعرف عليه وسوف يتم الحفاظ على المعلومات التي تم جمعها من الاستبيان في مكان آمن و لن يتم مشاركتها مع أي أحد و سيتم استخدامها فقط لغرض التحليل و نشر نتائج الدراسة و من ثم التخلص منها بالطريقة المناسبة.

**ملاحظه: استكمالك للاستبيان وإرساله يعني موافقتك مستنيره على المشاركة في الدراسة.**

**في حال وجود أي استفسار، يرجى التواصل على الإيميل:**

[**OQADHI@KSU.EDU.SA**](mailto:OQADHI@KSU.EDU.SA)

**شكرا لموافقتك على استكمال الاستبانة**

My name is Omaimah Qadhi a researcher from Collage of Nursing at King Saud University in Riyadh. I am conducting this research study aiming to assess and explore the attitude and perception of the healthcare providers towards learning and using sign language in communicating with deaf patient or who are with hearing impairments.

If you are a healthcare provider and work in government or private hospitals or work in primary health centers in Saudi Arabi, you are eligible to participate in this study.

For your information, participating in this study is voluntary and does not entitle the participant and type or rewards. **You always have to full right to opt out from the study at any time you feel so with no any obligations or consequences on you.
The collected data from this study will be securely stored and used for the purpose of this study only and will be discarded appropriately after disseminating of the study findings.**

**Answering the survey and submitting it, means you full awareness and agreement in participating.**

Shall you have any concerns, please don’t hesitate to contact the primary researcher at: [OQADHI@KSU.EDU.SA](mailto:OQADHI@KSU.EDU.SA)

Your cooperation is highly appreciated.

**This survey is a main part in the study that is aiming the**

**Section A: demographics**

1. **Gender:** الجنس
2. Female**. أنثى**
3. Male. **ذكر**
4. **Age:** العمر
5. 18-25 years
6. 26-33 years.
7. 34-41 years
8. Older than 42 years
9. **Clinical work experience at** الطبي الميداني الخبرة في العمل مدة
10. None. **لا يوجد**
11. Less than 5 years **أقل من خمس سنوات**
12. More than 5 years**أكثر من خمس سنوات**
13. **Status** الحالة
14. Student **طالب/ه**
15. Nurse **ممرض/ه**
16. Physician **طبيب/ه**
17. Other healthcare providers (specify) **ممارس صحي حدد ......................**
18. **Work area** مكان العمل
19. Outpatient **العيادات الخارجية**
20. Inpatient **التنويم**
21. Primary care center **مركز رعاية أولية**
22. **Mother language** اللغة الأم
23. Arabic **العربية**
24. English**الإنجليزية**
25. Other (specify) **أخرى حدد ......................**
26. **Education level:** مستوى التعليم
27. Diploma in nursing **دبلوم في التمريض**
28. Bachelor in nursing**بكالوريوس في التمريض**
29. Master in nursing**ماجستير تمريض**
30. Other specify degree/major **أخرى حدد الدرجة و التخصص.....................................**
31. **Number of years since completing the program**المدة بعد إتمام برنامج الدراسة
32. I have no experience**. ليس لدي خبره**
33. Less than 2 years**أقل من سنتين**
34. Between 2- 5 years **بين السنتين و الخمس سنوات**
35. Between 5-10 years. **بين الخمس والعشر سنوات**
36. More than 10 years. **أكثر من عشر سنوات**

**Section B: Sing language.**

1. **Have you received any formal training in sign language?**

**هل سبق لك التدريب على استخدام لغة الإشارة؟**

- 1. Yes نعم
  2. No لا

1. **In your opinion, how important is it for healthcare providers to be able to communicate using sign language with patients who are deaf or have hearing impairments?**

**هل ترى من وجهة نظرك أنه من الضروري قدرة مقدمي الرعاية الطبية على التواصل باستخدام لغة الإشارة عند التعامل مع المرضى الصم أو ضعيفي السمع؟**

- 1. Very important مهم جدا
  2. Somewhat important نوعا ما مهم
  3. Not very importantليس مهم كثيرا
  4. Not at all important ليس مهم أبدا

1. **How often do you encounter patients who are deaf or people with hearing impairment in your work setting?**

كم مرة حصل أن كان لديك مريض من فئة الصم أو ضعاف السمع؟

- 1. Dailyيوميا
  2. Weekly أسبوعيا
  3. Monthly شهريا
  4. Rarelyنادرا
  5. Neverلم يحصل أبدا

1. **Do you feel comfortable communicating with a patient who uses sign language?**

**هل تشعر بارتياح عندما تتواصل مع شخص يستخدم لغة الإشارة؟**

- 1. Yesنعم
  2. Noلا
  3. Sometimesأحيانا

1. **What methods do you currently use to communicate with patients who are deaf or hard of hearing?**

**ماهي الطريقة التي تستخدمها حاليا إذا اضطررت للتواصل مع شخص أصم أو ضعيف سمع؟**

- 1. Writing on a notepadالكتابة على ورقه
  2. Lipreading قراءة الشفاه
  3. Asking family members or friends to interpret أستعين بصديق أو أحد أفراد عائلة المريض.
  4. Using a translation app on a smartphone or tablet أستخدم تطبيق للترجمة بواسطة الجوال او الجهاز اللوحي
  5. Other (please specify) أخرى حدد ....................................
  6. Not applicable (NA) لا ينطبق

1. **Do you think that knowing sign language would improve the quality of care that you provide to patients who are deaf or hard of hearing?**

**هل تعتقد أن معرفتك و فهمك للغة الإشارة سوف يحسن من جودة الخدمة التي تقدمها/تقدميها لمريض أصم أو ضعيف سمع؟**

- 1. Yesنعم
  2. Noلا

1. **Have you ever felt that a patient's healthcare was compromised due to a lack of communication resources for patients who use sign language?**

**هل سبق وشعرت أن صحة المريض الأصم أو من ضعيفي السمع تأثرت سلبا بسبب عدم توفر مصادر للتواصل معهم باستخدام لغة الإشارة؟**

- 1. Yesنعم
  2. Noلا
  3. Not sure لست متأكد/ه

1. **Do you believe that sign language is a unified language or that it varies by region or dialect?**

**هل تعتقد أن لغة الإشارة موحده أو أنها تختلف حسب المنطقه و اللهجه؟**

1. Sign language is a unified language لغة الإشارة موحده و عامه
2. Sign language varies by region or dialect تختلف لغة الإشارة بحسب المنطقه أو اللهجه
3. Not sure لست متأكد/ه
4. **If you believe that sign language varies by region or dialect, do you think this impacts communication with patients who are deaf or hard of hearing?**

**إذا كنت تعتقد بإختلاف لغة الإشارة حسب المنطقه و اللهجهّ فهل تعتقد أن ذلك قد يؤثر على عملية التواصل مع المرضى الصم أو من ضعاف السمع؟**

1. Noلا
2. Not sure لست متأكد/ه
3. **What barriers, if any, do you think exist to learning and using sign language in your work setting?**

**إذا تعتقد أن هناك عوائق في مقر عملك، تعيق من تعلم و إستحدام لغة الإشارة؟**

- 1. Lack of time to learn عدم توفر الوقت
  2. Lack of resources (e.g. teachers, materials) عدم توفر مصادر (معلم، مواد تعليم )
  3. Lack of support from management or colleaguesإنعدام دعم الإداره أو الكليات
  4. Other (please specify) أخرى حدد..........................................................

1. **How would you rate the availability of sign language interpretation services in your work setting?**

**كيف تقيم مستوى توفر خدمات ترجمة لغة الإشارة في مقر عملك؟**

- 1. Excellentممتاز
  2. Goodجيد
  3. Fairلا بأس بها
  4. Poorضعيف

1. Non-existentلا توجد
2. **Would you be interested in receiving training in sign language?**

**هل لديك الرغبة الحصول على تدريب خاص بلغة الإشارة؟**

- 1. Yesنعم

1. Noلا
2. **If yes, what form of training would you prefer?**

**إذا كانت الإجابة بنعم، ماهي طريقة التدريب التي ترغب بها؟**

- 1. In-person classesوجها لوجه
  2. Online classesعن بعد
  3. Self-study materials (e.g. books, videos) تعليم ذاتي (كتاب أو فيديو)
  4. Other (please specify) أخرى حدد....................................

1. **In which language would you prefer to learn sign language?**

**بأي لغة تفضل أن تتعلم بها لغة الإشارة؟**

- 1. Arabicعربي
  2. Englishإنجليزي
  3. Other (please specify)أخرى حدد..............

**The End**
